# Supplementary material for: Ataluren for the Treatment of Usher Syndrome 2A Caused by Nonsense Mutations
Source: Int J Mol Sci. 2019 Dec 12;20(24):6274. doi: 10.3390/ijms20246274 (PMC6940777; doi:10.3390/ijms20246274)
Supplement: Supplementary file 1 [file ijms-20-06274-s001.pdf]

International Journal of
Molecular Sciences

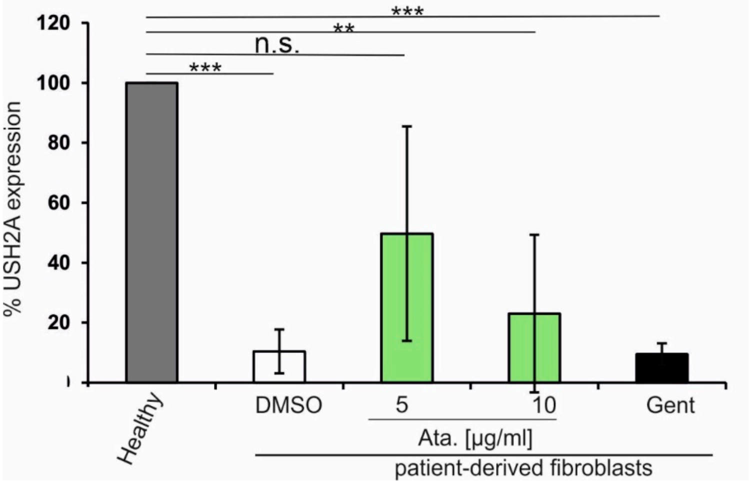

Figure S1. USH2A protein levels of untreated and TRIDS-treated patient-derived cells relative to healthy donor cells.

We calculated the relative USH2A expression levels in patient-derived cells versus healthy control fibroblasts. The band intensity for USH2A (Figure 3A) of control fibroblasts was set to 100%. A significant decrease of USH2A protein levels were detected in patient-derived fibroblasts treated with DMSO, 10 µg/µL Ataluren-treated or Gentamicin (Genta) compared to healthy control cells. No significant differences in protein levels were observed between healthy control cells and patient-derived cells treated with 5 µg/ml Ataluren. $p < 0.001$(***), $p < 0.01$ (**).

Table S1. Summary of the number of ciliated cells in patient-derived cells and healthy donor cells following TRID-treatment.

Cell Line	Treatment	Total Number	Ciliated Cells	Unciliated Cells
**Healthy donor** (*n* = 4)	Untr.	198	149	49
	DMSO	221	153	68
	Gentamicin	205	133	72
	Ataluren	180	133	47
**Patient-derived** (*n* = 4)	Untr.	97	51	46
	DMSO	100	50	50
	Gentamicin	93	35	58
	Ataluren	119	82	37
